# Development of Polyvinylidene Fluoride Membrane by Incorporating Bio-Based Ginger Extract as Additive

**DOI:** 10.3390/polym12092003

**Published:** 2020-09-03

**Authors:** Afrillia Fahrina, Nasrul Arahman, Sri Mulyati, Sri Aprilia, Normi Izati Mat Nawi, Aqsha Aqsha, Muhammad Roil Bilad, Ryosuke Takagi, Hideto Matsuyama

**Affiliations:** 1 Department of Chemical Engineering, Universitas Syiah Kuala, Banda Aceh 23111, Indonesia; afrilliafahrina26@gmail.com (A.F.); sri.mulyati@unsyiah.ac.id (S.M.); sriaprilia@unsyiah.ac.id (S.A.); 2 Doctoral Program, School of Engineering, Universitas Syiah Kuala, Jl. Syeh A. Rauf, No. 7., Banda Aceh 23111, Indonesia; 3 Graduate School of Environmental Management, Universitas Syiah Kuala, Jl. Tgk Chik Pante Kulu No. 5, Darussalam, Banda Aceh 23111, Indonesia; 4 Research Center for Environmental and Natural Resources, Universitas Syiah Kuala, Jl. Hamzah Fansuri, No. 4, Darussalam, Banda Aceh 23111, Indonesia; 5 Atsiri Research Center, Universitas Syiah Kuala, Jl. Syeh A. Rauf, Darussalam, Banda Aceh 23111, Indonesia; 6 Chemical Engineering Department, Universiti Teknologi PETRONAS, Seri Iskandar, Perak 32610, Malaysia; normi_16000457@utp.edu.my (N.I.M.N.); aqsha@utp.edu.my (A.A.); mroil.bilad@utp.edu.my (M.R.B.); 7 HiCoE-Center for Biofuel and Biochemical Research (CBBR), Institute for Self-Sustainable Building, Seri Iskandar, Perak 32610, Malaysia; 8 Research Center for Membrane and Film Technology, Department of Chemical Science and Engineering, Kobe University, Rokkodai-Cho 1-1, Nadaku, Kobe 657-0000, Japan; takagi@harbor.kobe-u.ac.jp (R.T.); matuyama@kobe-u.ac.jp (H.M.)

**Keywords:** antibiofouling, antimicrobial additive, bio-based membrane, ginger extract, membrane fouling

## Abstract

Biofouling on the membrane surface leads to performance deficiencies in membrane filtration. In this study, the application of ginger extract as a bio-based additive to enhance membrane antibiofouling properties was investigated. The extract was dispersed in a dimethyl acetamide (DMAc) solvent together with polyvinylidene fluoride (PVDF) to enhance biofouling resistance of the resulting membrane due to its antibiotic property. The concentrations of the ginger extract in the dope solution were varied in the range of 0–0.1 wt %. The antibacterial property of the resulting membranes was assessed using the Kirby Bauer disc diffusion method. The results show an inhibition zone formed around the PVDF/ginger membrane against *Escherichia coli* and *Staphylococcus aureus* demonstrating the efficacy of the residual ginger extract in the membrane matrix to impose the antibiofouling property. The addition of the ginger extract also enhanced the hydrophilicity in the membrane surface by lowering the contact angle from 93° to 85°, which was in good agreement with the increase in the pure water flux of up to 62%.

## 1. Introduction

Fouling in membrane filtration can occur due to the attachment or adsorption of foulant (organic/inorganic materials) contained in feed solution on the top of the membrane surface and within the membrane pores [1]. The membrane fouling phenomena is strongly affected by the interactions between foulant and membrane materials, typically through surface hydrophobic–hydrophobic interaction. Therefore, although pristine polymeric-based membranes are superior in mechanical and flexibility properties, they are prone to foul because of their hydrophobic nature [2,3,4].

Biofouling is initiated by the deposition and growth of microorganisms on the membrane surface. Then, the microorganisms produce microbial substances and form colonies called biofilms [5,6]. The formation of biofilm on the membrane surface establishes the cake layer, which enhances the overall filtration resistance. Unlike the organic and inorganic types of membrane fouling, biofouling is much more complicated to deal with. It often results in permanent loss of permeability [7]. Physical and chemical treatments can be carried out to resolve the biofouling. However, physical treatment alone is insufficient to restore the membrane performance from the irreversible fouling, while aggressive chemical treatments using strong acidic or alkaline solutions can potentially damage the membrane material and thus shorten membrane lifetime [8]. Overall, biofouling phenomena increase the operational and capital costs of a membrane system due to the increase in energy consumption (for biofouling control) and membrane chemical maintenance [9,10]. Therefore, the method for effective control of biofouling must be implemented to prevent severe permeability loss.

To curb biofouling formation, some antimicrobial substances have been added on membrane matrices such as graphene oxide (GO), silver (Ag), hydrous manganese dioxide (HMO) and titanium dioxide (TiO_2_) [11,12,13]. Ronen et al. [14] blended a polysulfones (PES) membrane matrix with nanosilver, and the results showed that nanosilver facilitated bacterial disinfection on the membrane surface by up to 27% and, thus, maintained the permeate flux in a prolonged operation because of the delay on the biofilm formation. In a different experiment, Lee et al. [15] reported that a composite membrane blended with GO nanoplatelets enhanced membrane antifouling capability in membrane bioreactor application.

Bio-based materials or chemicals offer several advantages compared to the synthetic materials, such as renewability, sustainability, and cost effectiveness. Some of the natural derivative substances such as curcumin, vanillin, and piper betel extract have been used as antibiofouling agents due to their antimicrobial property [11,16]. Arthanareeswaran et al. [5] employed quorum sensing inhibition (QSI) from vanillin to improve the antibiofouling property of the PES membrane. The results show that vanillin could limit the formation of microbial biofilm and increase the water permeability of the membrane. Kumar & Arthanareeswaran [11] added an antimicrobial additive from nano curcumin to the polyethersulfone membranes and showed a decrease in microbial activity on the membrane surface and, at the same time, increased the resulting membrane hydrophilicity.

Ginger (*Zingiber officinale Roscoe*) is a plant widely used in medicine, pharmaceutical, and food industries. The extract of ginger contains active substances in the form of zingerone, shogaols, gingerols and volatile (essential) oils which potentially pose antimicrobial and anti-oxidant effects [17,18,19]. Previous research has shown that ginger extract has strong antibacterial and antifungal properties against *Escherichia coli*, *Salmonella typhi*, *Bacillus subtilis*, *Salmonella enteritidis* and *Staphylococcus aureus* due to the presence of 6, 8 and 10-gingerol main active compounds [17,20,21,22]. Therefore, the employment of 6, 8 and 10-gingerol chemicals on membrane preparation has a potential to impose antibiofouling properties.

In the present work, we explored the application of pure ginger extract (*Zingiber officinale Roscoe*) as additive for fabrication of poly(vinylidene fluoride) (PVDF)-based membranes mainly for enhancing the antibiofouling property. The ginger extract additive offered few advantages apart from being bio-based, namely non-toxic, easy to handle, cost-effective and eco-friendly. The manufacturing of PVDF/ginger extract flat-sheet membrane was conducted via nonsolvent-induced phase separation, in which the ginger extract was incorporated into the dope solution by blending technique. The blending method is a simple process and has been used to enhance the resulting membrane properties [4]. The ginger extract was crushed through dry milling technique and blended in PVDF dope solution at various concentrations (*w*/*w* %). The antibiofouling of the PVDF membrane was investigated through an antibacterial test using the Kirby Bauer disc diffusion method against *Escherichia coli* and *Staphylococcus aureus* as representatives of the Gram-negative and the Gram-positive bacteria, respectively. The other impacts of ginger extract addition on PVDF membrane characteristics were also evaluated in terms of chemical composition, hydrophilicity, morphological structure, water permeation and mechanical/tensile strength.

## 2. Materials and Methods

### 2.1. Materials

PVDF polymer (molecular weight: 534,000 Da, Sigma Aldrich, USA) was employed as the main membrane material. Dimethyl acetamide (DMAc) (Wako Pure Chemical Industries, Japan) was used as a solvent. *Zingiber officinale roscoe* extract (purity 100%) (Sciyu bio. Tech, China) was utilized as an antibacterial additive. Deionized water (DI) was used as a nonsolvent for the solidification process. *Escherichia coli* (ATCC 25922) and *Staphylococcus aureus* (ATCC 25923) were used as representative of the Gram-negative and the Gram-positive bacteria.

### 2.2. Membrane Preparation

The nonsolvent-induced phase separation (NIPS) method was applied to fabricate asymmetric flat sheet membranes. Pure *Zingiber officinale roscoe* extract was ground firstly using a nano ball mill at 300 rpm speed over 30 h to facilitate easier blending in the dope solution. Then, different concentrations (wt %) of the ginger extract were dispersed in DMAc using an ultrasonicator for 30 min, followed by stirrer agitating for 5 min. The compositions of the dope solutions are shown in Table 1. A 16% (*w*/*w*) of PVDF polymer was added into the mixture and dissolved by stirring until homogeneous. The solution was degassed before being casted in a glass plate using a casting applicator (YBA-3, Yoshimitsu, Japan) with a wet gap of 200 μm. Subsequently, the casting plate was immediately immersed into a nonsolvent bath containing deionized water at temperature 25 °C for 20 min. The solidified membranes were finally stored in distilled water until further use.

### 2.3. Membrane Characterization

The functional groups in the membrane matrix were identified by an attenuated total reflectance (ATR) instrument (Thermo Scientific iD5 ATR–Nicolet iS5 FTIR spectrophotometer, Japan). A piece of 1 × 1 cm^2^ sample was analyzed using the ATR-FTIR at a wavenumber range of 400–4000 cm^−1^ to produce absorbance spectra.

Antibacterial activity was analyzed against *Escherichia coli* (ATCC 25922) to represent the Gram-negative and *Staphylococcus aureus* (ATCC 25923) to represent the Gram-positive bacteria through the Kirby Bauer disc diffusion method. The microbial solutions (*E. coli*/*S. aureus*) 0.5 Mac Farland were swiped onto solidified Mueller–Hinton agar under sterile conditions. The tested membrane samples were cut with a diameter of 5 mm and were sterilized under UV radiation (22 W, SUV-16 254 nm, AS ONE, Japan) for 30 min prior to antimicrobial testing. The sterile membranes were placed on the agar surface and incubated for 24 h at 37 °C. After 24 h, the inhibition zones that formed around the membrane sheets were observed and measured. Each sample was tested three times to ensure the reproducibility of the measurement.

The hydrophilicity on the membrane surface was measured by using a contact angle meter (Drop Master 300, Kyowa Interface Science Co., Japan). The amount of 1 μL of Milli-Q water was dropped onto 2 × 5 cm^2^ of the dry membrane samples. The measurements were taken five times and are presented as the average value with standard deviation.

The surface and cross-section membrane morphologies were visualized by using scanning electron microscopy (SEM; JSF-7500F, JEOL Co., Ltd., Japan) at 5.0 kV. Small pieces of membrane samples were freeze-dried (FD-1000, Eyela, Japan) and fractured in liquid nitrogen for preparing the cross-section sample. All specimens were sputtered by an osmium coater (Neoc-STB, Meiwafosis Co., Ltd., Japan) to impose conductive property on the SEM samples.

The mechanical property of the membranes was evaluated with a tensile test instrument (Autograph AGS-J, Shimadzu Co., Japan) according to the ASTM D 638-14 standard method. A sample with dimensions of 40 × 4 mm^2^ was pulled between two holders at the tension rate at 20 mm/min. Each sample was tested four times, and the data are presented as average and standard deviation. The thickness of the membrane sample was also measured with a micrometer tool (MCD130-25, Niigataseiki Co., Japan).

The permeation performance of membranes was characterized by the pure water flux (Equation (1)). A sheet of membrane with effective area of 9.075 cm^2^ was placed in a crossflow filtration cell, as described previously [3,23]. Distilled water feed was pumped into the filtration cell using a peristaltic pump (Watson Marlow, UK) with rotational speed 25 rpm. The solution inside the module was pressurized at trans-membrane pressure (TMP) of 1.0 bar, and the permeate was collected at intervals of 10 min until reaching a constant flux. Before conducting the pure water permeation, all membranes were pre-compacted for 1 h under the same pressure using DI water as the feed.

(1) $$J=\frac{V_{p}}{A_{m}\times t_{f}}$$

where, J is the pure water flux (L/m^2^·h or LMH); V_p_ permeate volume (L); A_m_ membrane effective area (m^2^) and t_f_ filtration time (h).

## 3. Results and Discussion

### 3.1. Characterization of the Ginger Extract

**Figure 1:**
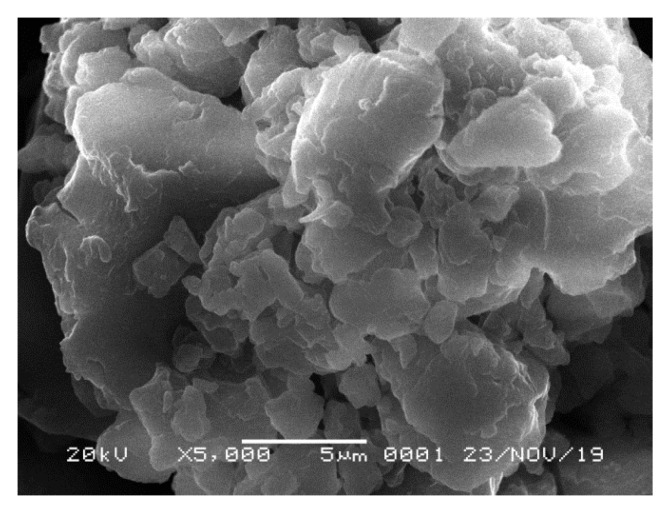
SEM image of the ginger extract used as membrane formation additive.

The morphology of ginger extract can be seen from the SEM images shown in Figure 1. The images depict that the ginger particles had heterogeneous shape and variable sizes. Some agglomerations were clearly seen because the particles were used in powder form. The agglomerates were expected to be dissolved in the dope solution, as observed by the formation of homogeneous solution without any obvious settlement after the mixing. The milling process somehow eased the dissolution of the ginger extract in the dope solution.

**Figure 2:**
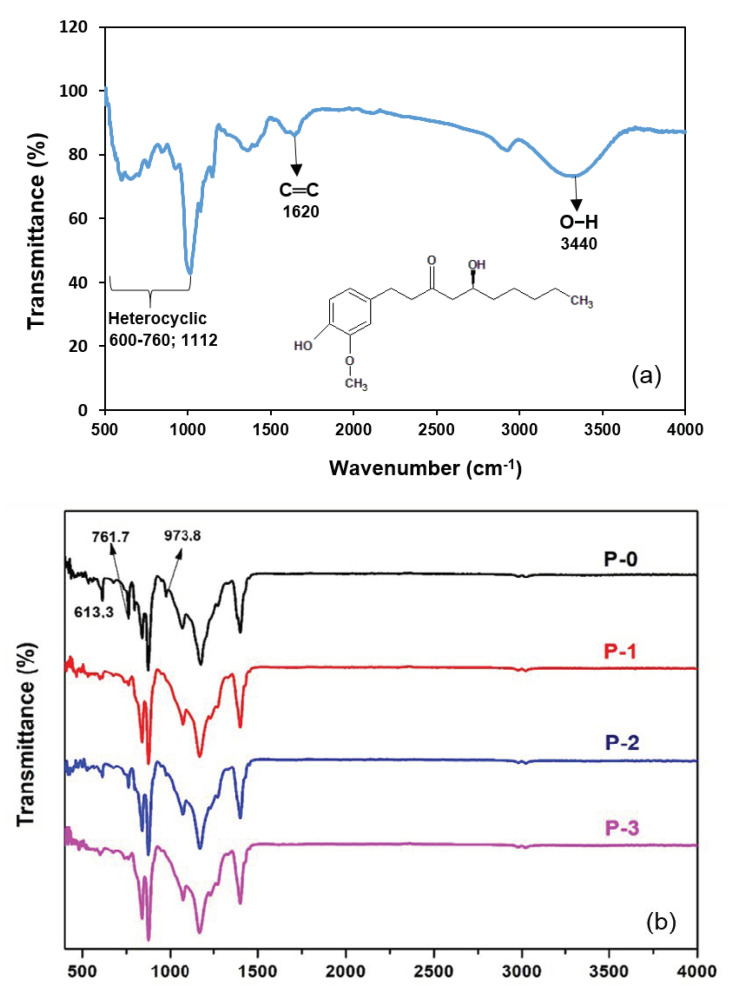
IR spectra of (**a**) the ginger particles and (**b**) the prepared membranes.

Figure 2a shows that an aromatic –OH group was observed at 3440 cm^−1^ with wide and strong stretching vibration [11]. The peaks at 1620 cm^−1^ and 1344 cm^−1^ indicate aromatic –C═C– and C–H bands of methyl rock, respectively. Heterocyclic compounds from active components of ginger such as flavonoids and alkaloids were represented by peaks at 600–760 cm^−1^ and 1112 cm^−1^ [18].

Figure 2b shows the IR spectra of membranes to identify chemical compounds present in nascent and modified PVDF membranes. Successively, the functional groups of CF_2_ deformation, vibration, and CH_2_ stretching, characteristic of the PVDF membrane, are shown at wavenumbers of 1183, 1407 and 2982 cm^−1^, respectively [24]. Some peaks at 613.3, 761.7 and 973.8 cm^−1^ indicated α-phase vibration of the PVDF C−F bond appearing only in the P-0 membrane. For the modified membranes with the addition of ginger (P-1; P-2; P-3), the C−F bond depressed probably due to an interaction between the C−F bond from PVDF with an aromatic structure of the ginger phenolic compound. The presence of heterocyclic-associated compounds in the membranes made with ginger extract additive suggests that some of those compounds reside in the membrane matrix, which are then expected to impose antimicrobial properties.

### 3.2. Membrane Morphological Structure

**Figure 3:**
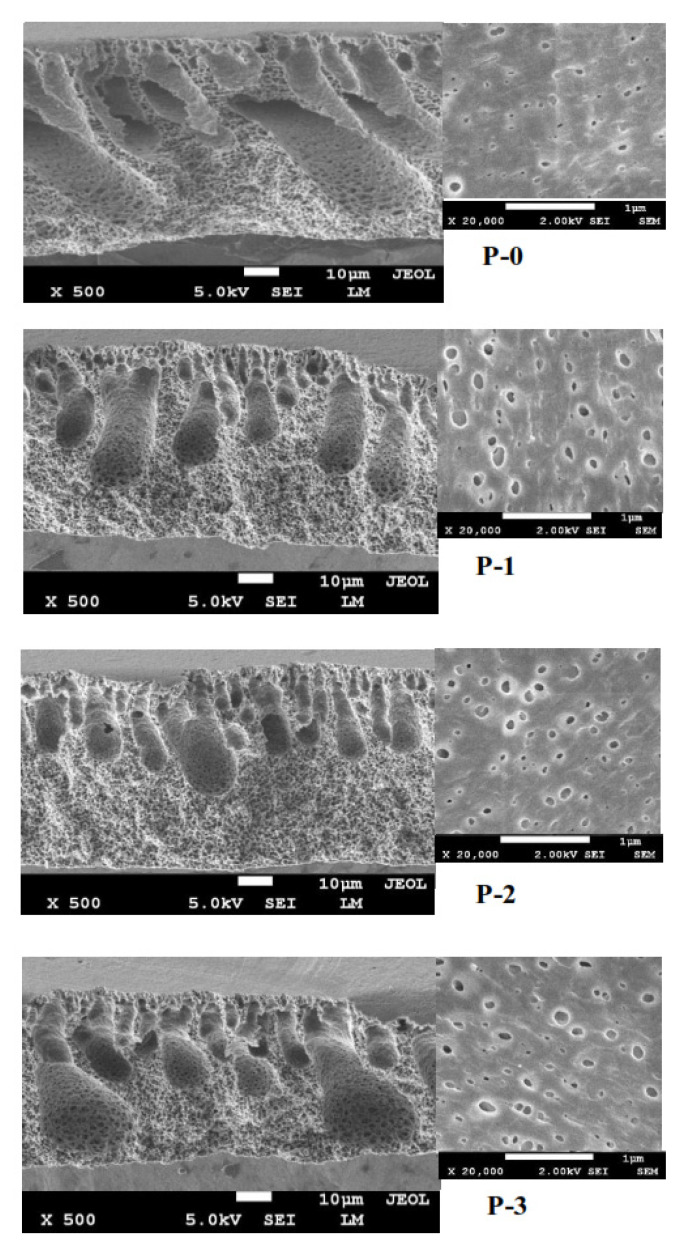
Membrane morphological structure on the surface and cross-section.

Figure 3 shows that the resulting membranes had asymmetric structures with a thin, dense layer and macrovoid in the sub-layer. The nascent and modified membranes had no significant change in the surface morphology. However, the cross-section morphology suggests that the membranes exhibited dissimilarity in the structures indicated by the transformation of membrane structure after loading of ginger extract. The nascent PVDF membrane had almost no macrovoid in the support layer. Nevertheless, after the addition of ginger, macrovoid grew in the bottom side of modified membranes (see cross-section images in Figure 3).

The increase in the ginger extract loading in the dope solution led to the increasing number and size of macrovoid, as shown in P-2 and P-3 morphological structures (Figure 3). The change of the membrane structure had a strong correlation with the thermodynamic and kinetic aspects between polymer solution and coagulation bath [24,25]. The formation of macrovoids was induced by the quicker exchange of solvent and nonsolvent during the phase inversion due to the presence of hydrophilic ginger [1,26]. The instantaneous demixing resulted in the formation of a sponge-like structure/morphology, which promotes porosity [27,28], as well as filtration performance, as discussed in Section 3.4.

### 3.3. Antibiofouling Property

**Figure 4:**
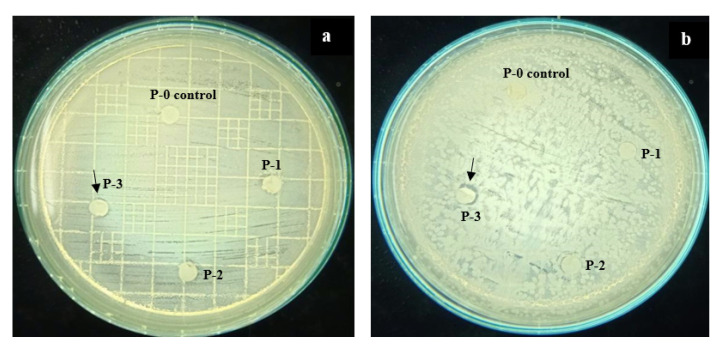
Inhibition zone of nascent and modified PVDF membranes against *E. coli* (**a**) and *S. aureus* (**b**).

Figure 4 shows that the P-0 as the control membrane did not show any inhibition zones on both of the petri dishes. In fact, the community of *S. aureus* grew very well around and on the membrane sample, likely because of the hydrophobic nature of the substrate as a good support for bacterial growth (Section 3.4) which facilitates adhesion of the cell wall.

The presence of the ginger extract at concentrations of 0.01–0.1 wt% in the dope solution imposed bacterial inhibition properties against both *E. coli* (Gram-negative) and *S. aureus* (Gram-positive). The phenolic compound contained in ginger extract has an aromatic structure and was employed as an anti-bacterial agent which demonstrated an obvious impact on imposing antimicrobial properties. The formation of an inhibition zone against *E. coli* was more pronounced than the inhibition zone for the *S. aureus*. The same result was also reported by Mesomo et al. [20]. However, all of the membranes generally showed slight inhibition. It could be explained as, during membrane preparation, blending and phase inversion, some parts of the aromatic structure might interact with PVDF structure (as confirmed in IR spectra, Figure 2) and led to opening of the cyclic aromatic structure and thus reduced the anti-bacterial effect of the ginger extract. Another plausible explanation is the low concentration of antimicrobial compound residing on the membrane matrix due to excessive leaching during the phase inversion. Quantitative analysis on the leaching will be the topic of a future study.

### 3.4. Membrane Surface Hydrophilicity and Pure Water Permeation

**Figure 5:**
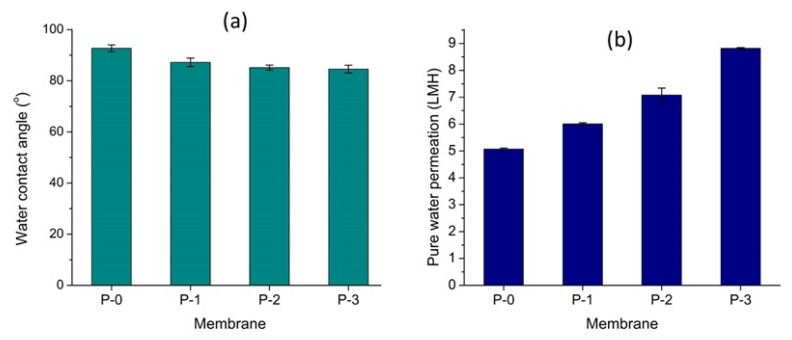
The effect of the ginger extract loading on the water contact angle (**a**) and pure water permeation (**b**) of the resulting membranes.

The improvement of PVDF membrane hydrophilicity after the addition of ginger extract is presented in Figure 5a. The water contact angle of the PVDF membrane decreased from 92.69° to 84.56° with the increment of ginger concentrations in the dope solution from 0 to 0.1% *w*/*w*. The loading dosage of the ginger extract correlated well with the membrane hydrophilicity. The biofilm produced by bacteria contains hydrophobic substances such as lipids and protein [29]. The increase in the hydrophilicity in the membrane surface was then expected to reduce the adhesion of microbial products [30]. Moreover, the presence of oxygen-containing functional groups, such as hydroxyl (−OH), carboxyl (−COOH) and epoxy groups, is well-known to improve the membrane hydrophilicity [31,32] because it creates strong intramolecular dipole moments. Therefore, hydroxyl (−OH) groups in the ginger extract-based chemicals were expected to enhance the membrane hydrophilicity.

The increase in surface hydrophilicity correlated well with pure water permeance (Figure 5b). It showed the increase in water permeation with the decreasing of water contact angle values. In other words, the enhancement of hydrophilicity in PVDF membrane surfaces was proven to enhance penetration of water molecules through the membrane pores [13,31]. The hydrophilic property of the ginger extract membrane facilitated pore-wetting and enhanced the pore numbers available for water permeation. In this case, significant increment of water permeance is shown in Figure 5b. The clean water permeance increased from 5.07 to 8.82 LMH for the plain PVDF membrane and the one loaded with 0.01 wt% of the ginger extract, corresponding to an increment of 62%.

### 3.5. Mechanical Property

**Figure 6:**
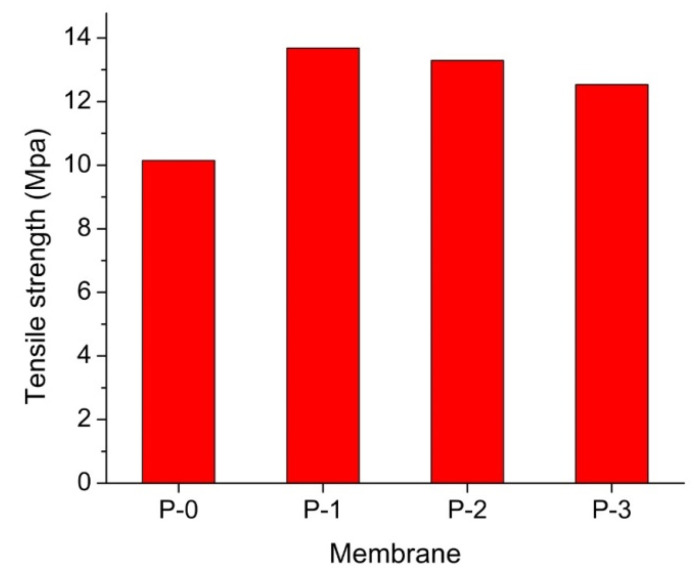
The effect of the ginger extract loading on the mechanical strength of the resulting membrane.

Figure 6 demonstrates that the addition of the ginger extract-loaded membranes posed higher tensile strength in comparison to the plain PVDF membrane. The mechanical property of the membranes is an important parameter to investigate membrane endurance for filtration application. The interaction between PVDF chains and the structures of the molecules contained in the extract increased compatibility and strengthened the polymer matrix of the resulting membranes [31,33]. As confirmed from FTIR in Figure 2a, the welding between the PVDF chain and aromatic groups of ginger extract potentially happened through hydrophobic–hydrophobic interaction, called as London dispersion force. The C−F groups of PVDF interacted with C=C groups of phenolic compound of the ginger extract.

The attraction force materialized between nonpolar molecules or the hydrophobic segments of the molecules. Therefore, all membranes loaded with the ginger extract membranes showed higher mechanical strength. The same result was also reported by Lin et al. (2020) [34] that ascribed the enhanced mechanical strength to the interaction between PVDF and styrene molecule. A steep increment from the plain PVDF membrane to the one loaded with 0.01 wt % was observed. Further enhancement of the ginger extract loading led to a slight decrease in the tensile strength. It can be explained by the formation of macrovoid in the membrane support layer leading to weakening and decreasing membrane tensile strength at higher loadings of the ginger extract in the dope solution [35,36].

## 4. Conclusions

Overall results show that the use of ginger extract is effective to enhance PVDF-based membrane properties and their hydraulic performance. Loading of the ginger extract in the polymer solution for membrane fabrication imposes antibiofouling properties on the resulting membranes, as shown by the growth inhibition zone formation around ginger extract-loaded membranes against both *E. coli* and *S. aureus*. The ginger extract additive promoted formation of macrovoid in the membrane sub-layer. The surface hydrophilicity of the membranes was also enhanced, as shown by the decrease in water contact angle from 93° to 85° with the increment of the ginger extract loading (up to 0.1 wt %) thanks to the presence of hydroxyl groups (−OH). It also showed good agreement to the value of pure water permeation with up to 62% increment. Furthermore, the addition of the ginger extract also enhanced membrane mechanical properties thanks to the good compatibility between PVDF polymer and the ginger extract.

## Figures and Tables

**Table 1:** Composition of dope solution for membrane fabrication.

Membrane	PVDF (wt %)	Ginger (wt %)	DMAc (wt %)
P-0	16	0	84.00
P-1	16	0.01	83.99
P-2	16	0.05	83.95
P-3	16	0.1	83.90
